# An ion‐paired moxifloxacin nanosuspension eye drop provides improved prevention and treatment of ocular infection

**DOI:** 10.1002/btm2.10238

**Published:** 2021-06-22

**Authors:** Aditya Josyula, Revaz Omiadze, Kunal Parikh, Pranjali Kanvinde, Matthew B. Appell, Pratikkumar Patel, Hiwa Saeed, Yogesh Sutar, Nicole Anders, Ping He, Peter J. McDonnell, Justin Hanes, Abhijit A. Date, Laura M. Ensign

**Affiliations:** ^1^ The Center for Nanomedicine, The Wilmer Eye Institute Johns Hopkins University School of Medicine Baltimore Maryland USA; ^2^ Department of Chemical and Biomolecular Engineering Johns Hopkins University Baltimore Maryland USA; ^3^ Department of Ophthalmology, The Wilmer Eye Institute Johns Hopkins University School of Medicine Baltimore Maryland USA; ^4^ Department of Biomedical Engineering Johns Hopkins University School of Medicine Baltimore Maryland USA; ^5^ Center for Bioengineering Innovation and Design Johns Hopkins University Baltimore Maryland USA; ^6^ Department of Pharmacology and Molecular Sciences Johns Hopkins University School of Medicine Baltimore Maryland USA; ^7^ Department of Pharmaceutical Sciences, The Daniel K. Inouye College of Pharmacy University of Hawaii Hilo Hawaii USA; ^8^ The Sidney Kimmel Comprehensive Cancer Center at Johns Hopkins University Baltimore Maryland USA; ^9^ Department of Environmental Health Sciences Johns Hopkins University School of Medicine Baltimore Maryland USA; ^10^ Department of Neurosurgery Johns Hopkins University School of Medicine Baltimore Maryland USA; ^11^ Department of Tropical Medicine, Medical Microbiology and Pharmacology, John A. Burns School of Medicine University of Hawaii Manoa Honolulu Hawaii USA; ^12^ Department of Gynecology and Obstetrics and Division of Infectious Diseases Johns Hopkins University School of Medicine Baltimore Maryland USA

**Keywords:** antibiotic, conjunctivitis, eye drop, keratitis, mucoinert, mucus‐penetrating particles

## Abstract

There are numerous barriers to achieving effective intraocular drug administration, including the mucus layer protecting the ocular surface. For this reason, antibiotic eye drops must be used multiple times per day to prevent and treat ocular infections. Frequent eye drop use is inconvenient for patients, and lack of adherence to prescribed dosing regimens limits treatment efficacy and contributes to antibiotic resistance. Here, we describe an ion‐pairing approach used to create an insoluble moxifloxacin–pamoate (MOX–PAM) complex for formulation into mucus‐penetrating nanosuspension eye drops (MOX–PAM NS). The MOX–PAM NS provided a significant increase in ocular drug absorption, as measured by the area under the curve in cornea tissue and aqueous humor, compared to Vigamox in healthy rats. Prophylactic and treatment efficacy were evaluated in a rat model of ocular *Staphylococcus aureus* infection. A single drop of MOX–PAM NS was more effective than Vigamox, and completely prevented infection. Once a day dosing with MOX–PAM NS was similar, if not more effective, than three times a day dosing with Vigamox for treating *S. aureus* infection. The MOX–PAM NS provided increased intraocular antibiotic absorption and improved prevention and treatment of ocular keratitis, and the formulation approach is highly translational and clinically relevant.

## 1 INTRODUCTION

While eye drops are the dominant dosage form in the ophthalmic market, achieving effective intraocular drug delivery via eye drops is actually quite challenging.^1^ Tear production, reflexive blinking, and nasolacrimal drainage limit residence time, while formulation and drug properties can further limit the potential for the rapid intraocular drug absorption needed.^1^, ^2^, ^3^ Many pharmaceutical drugs are water‐soluble salts^4^, ^5^ and when dosed topically to the eye, water‐soluble drugs often show increased systemic absorption and rapid drug elimination.^6^ Low solubility drugs are often formulated as suspensions of particulates that must also traverse the ocular mucus barrier that protects the surface from allergens, pathogens, and debris.^7^, ^8^ Thus, regardless of the dosage form, intraocular drug absorption is low, necessitating frequent administration.^9^, ^10^ In the case of antibiotic eye drops, drops may be prescribed for use in treating bacterial keratitis and conjunctivitis every hour for the first 48 h and then once every 4 h until the infection is resolved.^11^ As the required number of doses per day increases, patient compliance, and thus, treatment efficacy, decreases.^12^, ^13^, ^14^ Antibiotic eye drop formulations that are more effective with less frequent dosing are needed.

Antibiotics are also used extensively for prevention of postsurgical ocular infection.^15^ Approximately, 40% to 80% of all endophthalmitis cases occur after cataract surgery, with *Staphylococcus* species being the most common causative agent.^16^, ^17^ Both eye drops and intracameral injections have been studied for the prevention of postcataract surgery endophthalmitis, and there is evidence to suggest that using eye drops in addition to injections has the lowest risk of infection.^18^ Fluoroquinolones show broad spectrum efficacy in treating bacterial conjunctivitis and keratitis,^19^ and thus, are often used for preventing postsurgical infections.^18^ Moxifloxacin hydrochloride (MOX) is often the drug of choice, as it has been shown to have higher intraocular bioavailability compared to other fluoroquinolones.^3^, ^20^ However, overall limitations in drug absorption with eye drops mean that prophylactic antibiotic drops still must be used three times or more per day.^21^, ^22^

Various strategies have been employed for increasing intraocular drug penetration with topical dosing.^23^ One common approach is the addition of viscosity enhancing materials or the use of creams and ointments, which have issues with messiness and blurring vision.^2^ Penetration enhancers may also be used, though frequent and chronic dosing can lead to ocular irritation and toxicity.^24^ An alternative approach that has been demonstrated for a wide variety of mucosal surfaces, including the eye, female reproductive tract, gastrointestinal tract, and airways is to formulate nanoparticles with mucoinert surface coatings.^25^, ^26^ These so‐called mucus‐penetrating particles (MPP) are nonadhesive to the sticky mucin proteins and small enough in size to fit through the net‐like pores, leading to enhanced delivery of small‐molecule drugs and nucleic acids alike.^25^ In the context of eye drops, MPP provide rapid and enhanced intraocular drug absorption, leading to the Food and Drug Administration (FDA) approval of two loteprednol etabonate (LE)‐based products for treating ocular inflammation and pain.^7^ Here, we describe an approach for forming an insoluble ion‐paired complex of MOX with pamoic acid that can be formulated into a mucoinert nanosuspension (MOX–PAM NS). We demonstrate here that the MOX–PAM NS provides equivalent or better prevention and treatment of ocular *Staphylococcus aureus* infection with once a day dosing compared to three times a day dosing with the commercial formulation, Vigamox®. We anticipate that such a reduction in dosing frequency while maintaining therapeutic effect would have a positive impact on patient care and quality of life.

## 2 RESULTS

### 2.1 Nanosuspension formulation and characterization

**FIGURE 1:**
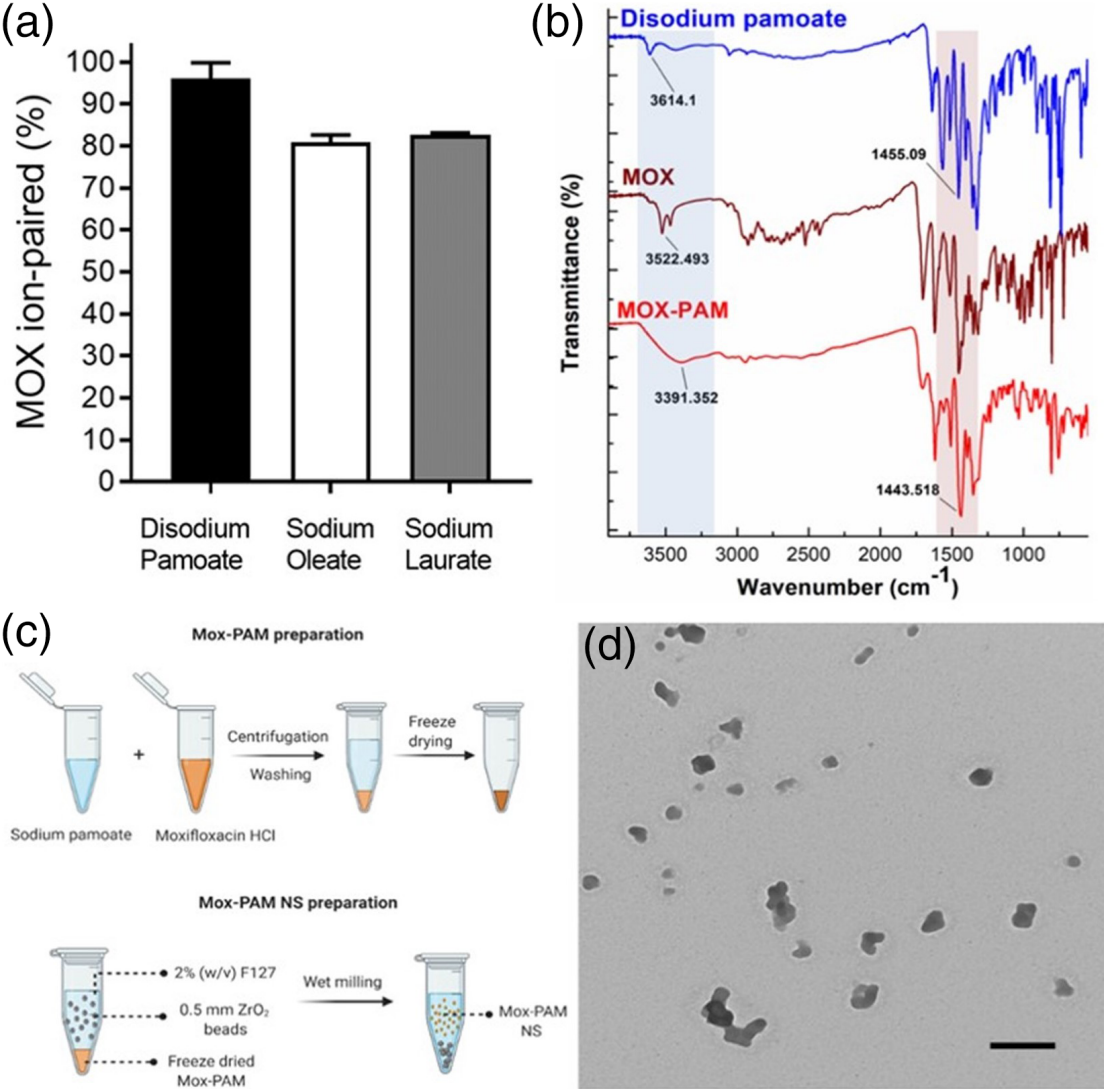
Water‐soluble anions electrostatically interact with water‐soluble moxifloxacin hydrochloride (MOX) to form insoluble complexes. (a) Disodium pamoate was the most efficient in ion pairing with MOX. Data expressed as mean ± SD, *n* = 3. (b) Fourier‐transform infrared (FTIR) spectrum of MOX‐pamoate (MOX–PAM) showed disappearance of the peak (3522.493) corresponding to the N─H group in MOX and shift in the peaks (3614.1 cm^−1^ and 1455.09 cm^−1^) corresponding to the ─OH of the carboxylic acid in disodium pamoate, indicating the formation of ion pair. (c) Schematic depicting the process to prepare MOX–PAM nanosuspension (MOX–PAM NS). (d) Transmission electron micrograph showing MOX–PAM NS (scale bar = 500 nm)

Pharmaceutical anionic ion‐pairing agents, sodium laurate, sodium oleate, and disodium pamoate, were able to convert hydrophilic and cationic MOX into hydrophobic ion pairs after simple mixing. The disodium pamoate showed highest ion‐pairing efficiency (>95%) among the tested ion‐pairing agents at the concentrations tested (Figure 1(a)). The Fourier‐transform infrared (FTIR) spectrum of MOX–PAM showed suppression of the N─H stretching band and shifting of O─H stretch of carboxylic acid in MOX (Figure 1(b)), indicating the interaction between MOX and disodium pamoate to form MOX–PAM ion pair via electrostatic interaction. A schematic for the formation of MOX–PAM and formulation of the nanosuspension is shown in Figure 1(c). Using our previously described wet‐milling method, we successfully developed MOX–PAM NS with particle size of 233 ± 26 nm, average polydispersity index (PDI) of 0.15, and zeta potential value of −10.4 ± 0.37 mV (*n* = 3). Transmission electron microscopy (TEM) imaging of MOX–PAM NS showed near spherical morphology (Figure 1(d)), where the average particle size measured using TEM images was in good agreement with the Zetasizer measurements (198 ± 54 nm). Short‐term stability characterization over 2 weeks showed stable particle diameter when stored at room temperature, with a slight increase in size when stored at room temperature (Figure S1).

### 2.2 Topically administered MOX–PAM NS provides increased intraocular drug absorption compared to Vigamox

The ocular pharmacokinetics study in healthy rats showed that MOX–PAM NS provided rapid and increased delivery of MOX compared to Vigamox in the aqueous humor, with ~1.6‐fold greater *C*_max_ (Table 1). The cumulative drug exposure was significantly increased in animals treated with MOX–PAM NS, with AUC_0–24h_ values in aqueous humor and cornea ~1.7‐fold and ~4.4‐fold higher, respectively, compared to Vigamox‐treated animals (Table 1, *p* < 0.05).

**TABLE 1:** Pharmacokinetic parameters of topically applied Vigamox (0.5% w/v) or MOX–PAM NS (equivalent to 0.5% w/v MOX) in rats

Matrix	MOX–PAM NS			Vigamox		
	*C*_max_ (μg/g)	*t*_max_ (h)	AUC_0–24h_ (μg*min/g)	*C*_max_ (μg/g)	*t*_max_ (h)	AUC_0–24h_ (μg*min/g)
Cornea	106.5 ± 66.7	1.5	961.9 ± 269.2*	99.4 ± 31.4	0.25	555.2 ± 66.8
Aqueous	153.3 ± 41.7	0.25	780.6 ± 404.5*	98.5 ± 27.8	0.5	175.9 ± 27.6

*Note*: Data expressed as mean ± *SEM* (*n* ≥ 4 eyes per group).

Abbreviations: MOX, moxifloxacin; PAM, pamoate; MOX–PAM NS, moxifloxacin–pamoate nanosuspension.

* *p* < 0.05 when compared to Vigamox.

### 2.3 MOX–PAM NS is more effective than Vigamox for preventing and treating *S. aureus* infection

**FIGURE 2:**
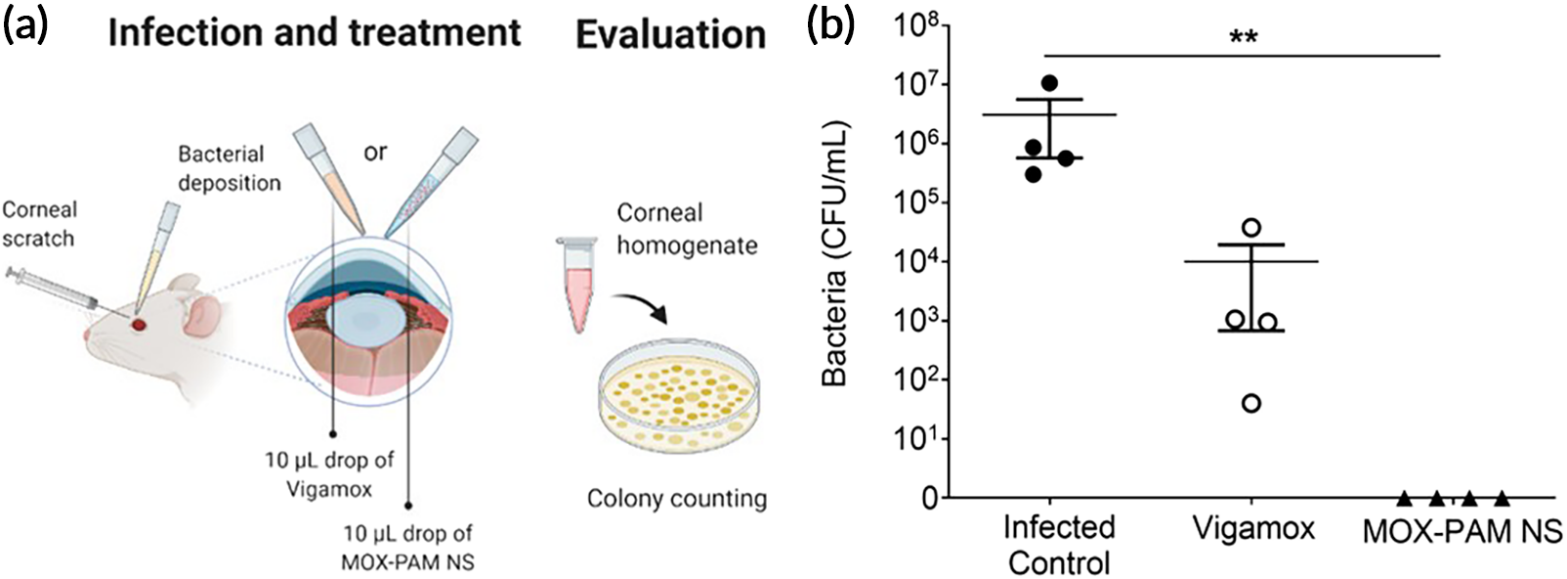
The moxifloxacin–pamoate nanosuspension (MOX–PAM NS) was more effective in preventing ocular *Staphylococcus aureus* infection. (a) To assess preventative efficacy, rats were inoculated topically with *S. aureus*, followed by either no treatment (Infected control), one 10 μl drop of Vigamox (0.5% w/v), or one 10 μl drop of MOX–PAM NS (equivalent to 0.5% w/v MOX). (b) Twenty‐four hours later, eyes were enucleated for the determination of the bacterial burden in corneal homogenates (n = 4 per group). Topical treatment with MOX–PAM NS completely prevented ocular infection, whereas Vigamox was only partially effective. Data expressed as mean ± SEM, ** *p* < 0.01

One of the most common uses of antibiotic eye drops is to prevent postsurgical infection. To assess efficacy in preventing infection, we topically administered a single drop of MOX–PAM NS or Vigamox (0.5% w/v MOX) immediately after *S. aureus* inoculation (Figure 2(a)). The corneal homogenates collected 24 h later showed 0.5 × 10^7^ CFU/ml for untreated infected control animals (Figure 2(b)). The animals treated with Vigamox showed ~2.7 log reduction in bacteria in the corneal homogenates compared to untreated animals (Figure 2(b), *p* < 0.05). In contrast, animals treated with MOX–PAM NS did not have any culturable bacteria in the corneal homogenates (Figure 2(b)) or in the corneal swabs that plated without dilution (Figure S2), suggesting complete prevention of infection. Histological evaluation showed that the corneas from animals treated with MOX–PAM NS looked normal, whereas the untreated control group showed infiltration of inflammatory cells (Figure S3).

**FIGURE 3:**
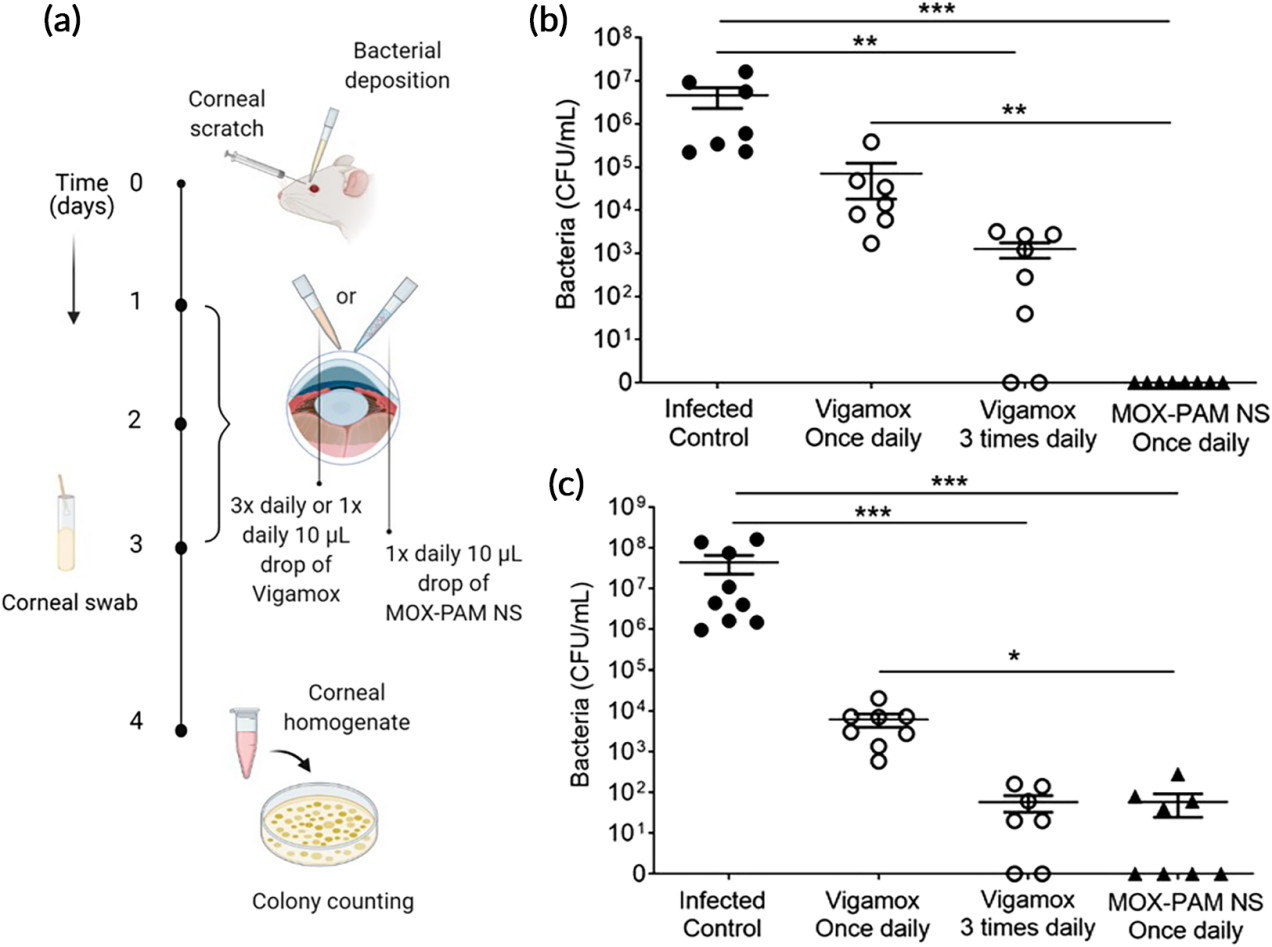
Once a day topical treatment with the moxifloxacin–pamoate nanosuspension (MOX–PAM NS) was more effective in treating ocular *Staphylococcus aureus* infection. (a) To assess therapeutic efficacy, rats were infected topically with *S. aureus* 24 h prior to initiating treatment. Rats received either no treatment (Infection control), once a day Vigamox (0.5% w/v), three times a day Vigamox, or once a day MOX–PAM NS (equivalent to 0.5% w/v MOX). (b) On the third day of treatment, corneal swabs were taken with a cotton‐tipped applicator for determination of bacterial load (*n* ≥ 7 per group). (c) Twenty‐four hours after the end of treatment (Day 4), the eyes were enucleated and were prepared for the determination of bacterial burden (*n* ≥ 7 per group). Once a day MOX–PAM NS had similar treatment efficacy as three times a day Vigamox. Data expressed as mean ± *SEM*, *p < 0.05; **p < 0.01; ***p < 0.001

We then assessed the comparative efficacy of MOX–PAM NS and Vigamox (0.5% w/v MOX) in treating established *S. aureus* corneal infection by starting treatment 24 h after high‐dose bacteria inoculation (Figure 3(a)). Vigamox is approved for three times a day dosing, so we compared three times a day dosing to once a day dosing. On the third day of treatment (Day 3 postinfection), the bacteria concentrations from the corneal swabs showed 4.3 × 10^6^ CFU/ml for the untreated infected control animals (Figure 3(b)). Once a day Vigamox provided ~1.8 log reduction (*p* < 0.05) and three times a day Vigamox provided ~3.5 log reduction (*p* < 0.01) compared to the untreated infected control animals (Figure 3(b)). In contrast, animals treated with once a day MOX–PAM NS did not have any culturable bacteria in the corneal swabs, a significant improvement over once a day Vigamox (Figure 3(b) *)*, *p* < 0.001). We then collected the cornea tissues 24 h after the last eye drop treatment (Day 4) for homogenization and determination of bacterial burden. The bacteria concentrations showed 5.5 × 10^6^ CFU/ml for the untreated infected control animals (Figure 3(c)). Once a day Vigamox provided ~3.8 log reduction (*p* < 0.05) and three times a day Vigamox provided ~5.8 log reduction (*p* < 0.001) compared to the untreated infected control animals (Figure 3(c)). Treatment with once a day MOX–PAM NS eye drop also provided ~5.8 log reduction, similar to three times a day Vigamox, and significantly more effective than once a day Vigamox (Figure 3(c), *p* < 0.05). The histological analysis showed significant infiltration of inflammatory cells in the untreated infected control corneas whereas three times a day Vigamox and once a day MOX–PAM NS‐treated animals had histologically normal looking cornea tissue (Figure S4).

## 3 DISCUSSION

About 70% of ocular infections are caused by bacteria.^19^, ^27^ In the United States, it was reported that there were 4 million cases of bacterial conjunctivitis and nearly 1 million clinic visits due to microbial keratitis per year.^28^, ^29^ *Staphylococcus* species are the most common causative agent for both conjunctivitis and keratitis, and topical fluoroquinolones are typically used for treatment.^19^ *Staphylococcus* species are also the most common pathogens that cause endophthalmitis, and thus, fluoroquinolone eye drops are routinely prescribed off‐label for preoperative and postoperative administration to prevent postsurgical endophthalmitis.^17^ Despite the overall low risk of postsurgical infection, the more than 2 million procedures per year in the United States make cataract surgery the most common cause of endophthalmitis.^17^, ^27^ Antibiotic eye drops are prescribed for use at least three times a day, and up to once every hour for severe infections. As the required number of doses per day increases, patient compliance, and thus, treatment efficacy, decreases. Issues with adherence can lead to sight‐threatening complications and potentially contribute to bacterial resistance. Antibiotic eye drop formulations that are more effective with less frequent dosing are needed to improve patient outcomes and quality of life and slow the development of bacterial resistance.

Similar to other exposed epithelial surfaces, the ocular surface is protected by mucus.^30^ Membrane spanning mucins form a dense glycocalyx that protects the conjunctival and corneal epithelium, while soluble mucins secreted by the goblet cells in the conjunctival epithelium are released into the tear film.^31^ Both the adhesive nature of mucins and the rapid turnover by blinking and tear secretion facilitate clearance of allergens, pathogens, and other debris. It has been demonstrated in multiple species that nanoparticles formulated with mucoinert surfaces (MPP) show improved topical ocular drug delivery.^7^, ^8^ In one study, LE was formulated as drug‐core nanoparticles coated with either Pluronic F127 (LE‐MPP) or sodium dodecyl sulfate (conventional particles, LE‐CP), or as microparticles (LE‐Micro). When evaluating intraocular bioavailability of LE in rabbits with a single topical dose, the LE‐MPP consistently provided higher drug levels in the cornea and the retina compared to LE‐CP or LE‐Micro.^7^ Similarly, a single dose of LE‐MPP 0.4% provided increased delivery of LE to various rabbit ocular tissues compared to Lotemax 0.5% suspension.^8^ Together, these observations supported the hypothesis that particles must be both mucoinert and small enough in size to avoid steric entrapment for increased topical drug absorption. However, the LE‐MPP formulation approach is suitable for water‐insoluble drugs, whereas the majority of drugs dosed as eye drops are water‐soluble salts.^4^, ^32^ When dosed topically to the eye, water‐soluble drugs often show increased systemic absorption and rapid drug elimination.^6^

Here, we described an approach for forming an insoluble ion‐paired complex of drug salts with commonly used pharmaceutical counter‐ions that can be formulated into mucoinert nanosuspensions for topical dosing. The mucoinert MOX–PAM NS provided increased intraocular drug absorption compared to Vigamox, including ~1.6‐fold increase in *C*_max_ in the aqueous humor, and ~1.7‐fold and ~4.4‐fold higher AUC_0–24h_ in the aqueous humor and cornea, respectively. While the relative drug concentrations in the rat eye were informative for understanding the improved efficacy with the MOX–PAM NS, it is difficult to compare the PK parameters to prior studies in rabbits and humans that reported lower intraocular moxifloxacin concentrations after dosing Vigamox.^3^, ^33^, ^34^, ^35^, ^36^ It is well known that corneal thickness varies considerably across the species, and the reported values of the average thickness of rabbit and human cornea are approximately ~2.2‐fold and 3.5‐fold higher than that of rat cornea.^37^, ^38^ Furthermore, the rats used here were albino, whereas it has been described that fluoroquinolones bind to melanin in the pigmented tissues in the eye.^39^ One additional factor to consider is that the rats used for PK experiments here had intact corneas, whereas it has been described that higher amounts of drug may penetrate into the eye when there is a surgical incision.^40^

Pamoate is a counter ion for several pharmaceutical drugs taken by the oral route and given as a sustained release intramuscular depot.^41^, ^42^ Furthermore, we recently described using pamoic acid as an ion‐pairing agent to formulate sustained‐release microcrystals that were injected in the subconjunctival space to provide protection of retinal ganglion cells.^43^ Pamoic acid did not negatively impact retinal ganglion cell survival in vitro at concentrations as high as 10 mg/ml.^43^ While encouraging, the safety of pamoic acid dosed topically would need to be demonstrated before use in an eye drop. Furthermore, while the short‐term stability data are encouraging, longer‐term characterization and, if required, additional optimization to ensure long‐term shelf stability would be an important next step in development.

Increasing drug absorption efficiency can lead to equivalent efficacy with fewer doses. Adherence to eye drop regimens is an often cited problem, and eye drops can only be effective when they are used as prescribed.^44^ Adherence is particularly a concern for elderly and pediatric patients, where reducing the required number of drops per day could significantly improve quality of life.^44^, ^45^ Better adherence in addition to increased and more effective drug concentrations can also circumvent issues with the development of bacterial resistance.^46^ In the case of LE‐MPP, the formulation was developed as a 1% suspension and approved in 2018 as the first twice‐daily ocular corticosteroid for post‐surgical inflammation and pain, where other prior products had to be dosed four times per day.^8^ The development of an antibiotic eye drop that has similar efficacy with once a day dosing compared to three times per day dosing would also be clinically impactful. Reformulation of a drug at the same concentration or lower than the approved concentration is a relatively straightforward path to clinical development.^47^ Importantly, this approach is amenable to a variety of other water‐soluble ionizable drugs used to treat glaucoma, dry eye disease, and inflammation, among others, suggesting broad potential impact.

## 4 MATERIALS AND METHODS

### 4.1 Materials

MOX (>99% purity) was purchased from LC Labs (Woburn, MA). Formalin, PAM, sodium oleate, sodium laurate, and sodium chloride were purchased from Sigma (St. Louis, MO). High‐performance liquid chromatography (HPLC)‐grade acetonitrile, sodium dihydrogen phosphate, and triethylamine were obtained from VWR International (Radnor, PA). Vigamox® (0.5% moxifloxacin eye drops, Alcon Laboratories Inc., Fort Worth, TX) was obtained from the Johns Hopkins Pharmacy. Zirconium oxide beads (0.5 mm in diameter) were purchased from Next Advance (Averill Park, NY). Sterile cell strainers (100 μm) were purchased from Fisher Scientific (Hampton, NH). Pluronic F127 (Poloxamer 407) was obtained as free samples from BASF Corporation (Tarrytown, NY). Tryptic soy agar, tryptic soy broth, sterile cotton‐tipped applicator, sterile disposable petri plates, and sterile phosphate‐buffered saline (PBS) and 20‐G needles were purchased from Thermo Fisher Scientific (Walthum, MA). *Staphylococcus aureus* (ATCC 25923) was obtained from ATCC (Manassas, VA). Proparacaine eye drops were obtained from Bausch and Lomb Inc. (Tampa, FL).

### 4.2 HPLC analysis of MOX

MOX was analyzed by HPLC using a previously reported method with minor modifications.^48^, ^49^ The HPLC apparatus consisted of a binary pump (Shimadzu, Burnsville, MN), a Shimadzu SPD‐20A UV–Vis detector (Shimadzu), and a prominence autosampler injector. Briefly, a stock solution of MOX was prepared in water and/or methanol, and the stock solution was further diluted with acetonitrile to obtain a concentration range of 1–10 μg/ml. Chromatographic separation was achieved using a Gemini C_18_ reversed phase column (150 × 4.6 mm, 3‐μm particle size). The mobile phase was composed of a 50:50 (v/v) mixture of acetonitrile and buffer containing 8.34 mM sodium dihydrogen phosphate, 0.7% v/v trimethylamine, and sufficient orthophosphoric acid to adjust to pH 2.5. The flow rate of the mobile phase was 1 ml/min. The column oven temperature was set at 45°C. MOX was monitored at 296 nm, and the retention time was 2.75 min. Data were analyzed using Lab solution integrator software 5.87 SP1.

### 4.3 Ion pairing

Hydrophobic ion‐pair complexes of MOX with sodium oleate, sodium laurate, and disodium pamoate were prepared using previously reported methods.^50^, ^51^, ^52^ Briefly, MOX (5 mg/ml, 11.41 mM), sodium oleate (11.41 mM), sodium laurate (11.41 mM), and disodium pamoate (PAM, 5.07 mM) were dissolved in ultrapure water. The PAM was prepared at half the molar concentration due to the presence of two carboxylic acid groups. MOX solution (0.5 ml) was mixed 1:1 with each of the ion‐pairing agents in an Eppendorf tube and vortexed at 1000 rpm for 5 min. The cloudy mixtures were then centrifuged at a speed of 10,000 rpm for 15 min to separate the solid complexes. The supernatant was withdrawn and transferred to an Eppendorf tube for the analysis of free MOX. The ion‐pair complexes were washed twice with water followed by centrifugation at 10,000 rpm for 15 min to remove any traces of free MOX and ion‐pairing agent. The MOX ion pair was freeze dried to remove any traces of water and stored in the refrigerator until further use. The MOX–PAM ion pair was further characterized by the FTIR using previously reported protocol.^53^

### 4.4 Development and characterization of MOX–PAM NS

The MOX–PAM NS was formulated based on thorough formulation development using a wet bead‐milling method previously described.^54^, ^55^ The wet‐bead milling was carried out using a lab‐scale tissue homogenizer (TissueLyser LT, Qiagen Inc, Germantown, MD). Briefly, MOX–PAM (10 mg), 1.5 g of 0.5 mm zirconium oxide beads, and 1 ml of 2% (w/v) Pluronic F127 solution were added to a 1.5‐ml Eppendorf tube. The contents were milled for 2 h at a speed of 3000 oscillations per min in a cold room. The mixture was then passed through a 100‐μm cell strainer to isolate the milling beads. Particle size, PDI, and surface charge (ζ‐potential) of the MOX–PAM NS were measured using a Malvern Zetasizer Nano ZS (173° scattering angle; *n* = 3 runs averaged for each of three independent samples). For the particle size and PDI measurement, MOX–PAM was diluted 1:50 in ultrapure water and for the ζ‐potential measurement, MOX–PAM NS was diluted 1:40 in 10 mM NaCl (pH 7). The morphology of the MOX–PAM NS was determined by the TEM. Briefly, a drop of the MOX–PAM NS (1:20 dilution) was placed on the formavar/carbon coated copper grid (Electron Microscopy Sciences), and the excess fluid was gently blotted using Kimwipes, before drying overnight at the room temperature. The morphology of MOX–PAM NS was determined using Hitachi H7600 transmission electron microscope. Particle size was evaluated using ImageJ (version 1.53j) with *n* = 4 images with at least three particles per image. For stability experiments, *n* = 3 batches of particles were stored at room temperature or under refrigeration for 14 days. Particle size was measured for each batch on Days 1, 4, 7, 11, and 14 and averaged.

### 4.5 Animal ethics statement

All animals were cared for and experiments conducted in accordance with protocols approved by the Animal Care and Use Committee of the Johns Hopkins University, the ARVO Statement for the Use of Animals in Ophthalmic and Vision Research, and the NIH Guide for the Care and Use of Laboratory Animals. Male Sprague Dawley rats (age 6–8 weeks; Harlan Laboratories, Frederick, MD) were used for the pharmacokinetic and in vivo efficacy studies. In the future, studies will be conducted with equal amounts of male and female animals to assess potential sex‐specific differences in infection or treatment response.

### 4.6 Topical ocular pharmacokinetics

Rats were anesthetized and received a drop (10 μl) of Vigamox solution or MOX–PAM NS (equivalent to 0.5% w/v of MOX) in one or both eyes, where animals in each group were treated the same at all time points (*n* = 2 in the Vigamox group at 8 h; *n* = 3 in the Vigamox group at 15 min, 1.5 h, and 24 h; *n* = 4 in the MOX–PAM NS group at all time points). At 15 min, 1.5 h, 8 h, and 24 h, rats were euthanized, and ocular tissues (aqueous humor and cornea) were collected and weighed. The aqueous humor and corneas were stored at −80°C until further analysis by liquid chromatography tandem mass spectrometry (LC–MS/MS). Cornea tissue samples were homogenized in 200 μl of 1× PBS (pH 7.4) before extraction. The standard curve and quality control samples were prepared in 1× PBS as a surrogate matrix for all matrices. MOX was extracted from 15 μl of aqueous humor or tissue homogenate with 50 μl of acetonitrile containing 1 μg/ml of the internal standard, moxifloxacin‐d4. After centrifugation, the supernatant was then transferred into autosampler vials for LC–MS/MS analysis. Separation was achieved with an Agilent Zorbax XDB‐C18 (4.6 × 50 mm, 5 μm) column with water/acetonitrile mobile phase (40:60, v:v) containing 0.1% formic acid using isocratic flow at 0.6 ml/min for 3 min. The column effluent was monitored using an AB Sciex triple quadrupole™ 4500 mass‐spectrometric detector (Sciex, Foster City, CA) using electrospray ionization operating in positive mode. The spectrometer was programmed to monitor the following multiple reaction monitoring (MRM) transitions: 402.0 → 383.9 for moxifloxacin and 406.2 → 108.0 for the internal standard, moxifloxacin‐d4. Calibration curves for moxifloxacin were computed using the area ratio peak of the analysis to the internal standard by using a quadratic equation with a 1/*x* ^2^ weighting function using two different calibration ranges of 0.5–500 ng/ml with dilutions up to 1:10 (v:v) and 5–5000 ng/ml. Cornea tissue samples were then quantitated in ng/g as: nominal concentration (ng/ml) × initial dilution ([tissue weight (mg) + volume of solvent (μl)]/ tissue weight (mg)) × additional dilution (where applicable). Pharmacokinetic parameters were calculated from individual MOX concentration–time data using standard noncompartmental methods as implemented in Phoenix WinNonlin version 8.3 (Pharsight A Certara Company).

### 4.7 Ocular infection prevention and treatment studies

Prevention and treatment of ocular infection in rats was assessed using a previously described model with minor adjustments.^56^ *S. aureus* bacteria were propagated on nutrient agar plates followed by incubation at 37°C for 16 h. The bacterial colonies were collected and suspended in sterile PBS to obtain bacterial count of 1 × 10^8^ CFU/ml. Rats received general anesthesia prior to applying a drop of 0.5% proparacaine hydrochloride ophthalmic solution to the cornea. The operative eye was then scratched using a 20‐G needle. A 100 μl droplet containing 1 × 10^8^ CFU/ml of *S. aureus* was then placed on the ocular surface. After 10 min, the excess fluid was removed by gently touching a Kimwipe to the ocular surface.

For prevention studies, animals were divided into three groups (*n* = 7): (1) untreated infection control, (2) Vigamox, and (3) MOX–PAM NS. Immediately after *S. aureus* inoculation, Group 2 and Group 3 received 10 μl of Vigamox and MOX–PAM NS, respectively. After 24 h, corneal swabs were taken with a cotton‐tipped applicator and dipped into 0.5 ml of sterile nutrient broth for 30 min. Subsequently, 0.1 ml of the broth was directly streaked onto tryptic soy agar plates and incubated for 24 h at 37°C for qualitative determination of relative bacterial burden. The rats were euthanized, and eyes were enucleated and either prepared for histological evaluation (*n* = 3) or evaluated for bacteria concentration (*n* = 4). To determine bacteria concentration, cornea tissues were placed in sterile tryptic soy broth and homogenized using a Power Gen 125 homogenizer (Fisher Scientific) for 4 min. Samples were then centrifuged at 300 rcf for 5 min. The supernatant was then serially diluted 10–10,000‐fold, and 100 μl of each dilution was streaked onto tryptic soy agar plates. The agar plates were stored at 37°C for 16 h prior to colony counting. The bacterial burden in different treatment groups and infection control was determined by taking into account the dilution factor used for each sample.

For treatment studies, 24 h after *S. aureus* inoculation, rats were divided into four groups (*n* ≥ 7): (1) untreated infection control, (2) once a day Vigamox, (3) three times a day Vigamox, and (4) once a day MOX–PAM NS. The eye drop treatment in Groups 2–4 was continued for 3 days. On the third day of treatment, corneal swabs were taken with a cotton‐tipped applicator to determine the bacteria concentration. The cotton‐tipped applicator was dipped into sterile tryptic soy broth for 30 min. The broth was then serially diluted 10–10,000‐fold, and 100 μl of each dilution was streaked onto tryptic soy agar plates. One day after the last dose, eyes were enucleated and either prepared for histological evaluation (*n* = 3) or evaluated for bacteria concentration (*n* ≥ 7). To determine bacteria concentration, cornea tissues were placed in sterile tryptic soy broth and homogenized using a Power Gen 125 homogenizer (Fisher Scientific) for 4 min. Samples were then centrifuged at 300 rcf for 5 min. The supernatant was then serially diluted 10–10,000‐fold, and 100 μl of each dilution was streaked onto tryptic soy agar plates. The agar plates were stored at 37°C for 16 h prior to colony counting. The bacterial burden in different treatment groups and infection control was determined by taking into account the dilution factor used for each sample.

### 4.8 Histological evaluation

For histological evaluation, the rats were euthanized, eyes enucleated, and fixed in formalin for 24 h. The eyes fixed in formalin were embedded in paraffin, cross‐sectioned, and stained with H&E by the Johns Hopkins Reference Histology Laboratory for histological evaluation.

### 4.9 Statistical analysis

The bacteria concentration in different groups was analyzed by one‐way analysis of variance followed by Turkey's multiple comparison test. The differences were considered significant at *p* < 0.05.

## 5 CONCLUSION

Herein, we described a clinically relevant approach for formulating moxifloxacin for improved treatment and prevention of bacterial keratitis. Due to low ocular bioavailability and rapid clearance, antibiotic eye drops must be used multiple times per day to be effective. Longer lasting treatments could improve patient adherence and treatment outcomes, though the high water solubility of MOX makes formulation for more sustained drug delivery challenging. By forming an insoluble complex between MOX and pamoic acid (MOX–PAM) and using a nanomilling process in the presence of Pluronic F127, a mucus‐penetrating nanosuspension formulation (MOX–PAM NS) was produced. The MOX–PAM NS was more effective than Vigamox when a single drop was given at the time of *S. aureus* inoculation in rats. When used as a treatment that was initiated 24 h after bacterial inoculation, once a day MOX–PAM NS was as effective as three times a day Vigamox in treating ocular *S. aureus* infection in rats. An effective antibiotic eye drop that can be used less frequently can improve the prevention and treatment outcomes of ocular infections.

## AUTHOR CONTRIBUTIONS

**Aditya Josyula:** Data curation; formal analysis; investigation; methodology; writing‐original draft; writing‐review & editing. **Revaz Omiadze:** Investigation; methodology; writing‐review & editing. **Kunal Parikh:** Formal analysis; methodology; supervision; writing‐review & editing. **Pranjali Kanvinde:** Investigation; writing‐review & editing. **Matthew Appell:** Investigation; writing‐review & editing. **Pratik Patel:** Investigation; writing‐review & editing. **Hiwa Saeed:** Investigation; writing‐review & editing. **Yogesh Sutar:** Investigation; writing‐review & editing. **Nicole Anders:** Formal analysis; investigation; methodology; validation; writing‐original draft; writing‐review & editing. **Ping He:** Investigation; writing‐review & editing. **Peter McDonnell:** Funding acquisition; methodology; project administration; supervision; writing‐review & editing. **Justin Hanes:** Conceptualization; funding acquisition; project administration; supervision; writing‐review & editing. **Abhijit Date:** Conceptualization; data curation; methodology; supervision; writing‐original draft; writing‐review & editing. **Laura Ensign:** Conceptualization; data curation; formal analysis; funding acquisition; methodology; project administration; supervision; writing‐original draft; writing‐review & editing.

## CONFLICT OF INTERESTS

The mucus‐penetrating particle technology is licensed and in clinical development for ocular indications by Kala Pharmaceuticals. Justin Hanes is a founder of Kala Pharmaceuticals and serves as a consultant. Justin Hanes, Laura Ensign, and Johns Hopkins University own company stock. Under a licensing agreement between Kala Pharmaceuticals and the Johns Hopkins University, Laura Ensign, Justin Hanes, and the University are entitled to royalty distributions related to the technology. These arrangements have been reviewed and approved by the Johns Hopkins University in accordance with its conflict of interest policies. All other authors declare no potential conflict of interests.

## Supporting information

**Figure S1** Nanosuspension particle diameter over 14 days of storage at room temperature (RT) or under refrigeration (4°C) (*n* = 3 per group). Data shown as mean ± *SEM*. *p < 0.05 compared to the starting diameter on Day 0
**Figure S2**. Moxifloxacin‐pamoate nanosuspension (MOX–PAM NS) showed superior prophylactic efficacy against ocular infections. The corneal swabs from *Staphylococcus aureus* infected rats from different groups were directly applied onto agar plates. A representative image of the agar plates from each group is shown to compare the bacterial burden qualitatively
**Figure S3**. Histopathological evaluation of corneas obtained from untreated rats (infected control) or rats treated with Vigamox or MOX–PAM NS immediately after *Staphylococcus aureus* infection (images representative of *n* = 3 per group). Scale bar represents 50 μm and applies to all images.
**Figure S4**. Histopathological evaluation of corneas obtained from untreated rats (infected control) or rats that received treatment beginning 24 h after *Staphylococcus aureus* inoculation with once daily Vigamox, three times daily Vigamox or once daily MOX–PAM NS for 3 days (images representative of *n* = 3 per group). Scale bar represents 50 μm and applies to all images

## Data Availability

The main data supporting the findings of this study are available within the paper and its Supplementary information. The associated raw data are available from the corresponding author on reasonable request.
