# Supplementary material for: An ion‐paired moxifloxacin nanosuspension eye drop provides improved prevention and treatment of ocular infection
Source: Bioeng Transl Med. 2021 Jun 22;6(3):e10238. doi: 10.1002/btm2.10238 (PMC8459599; doi:10.1002/btm2.10238)
Supplement: Supplementary file 1 — Figure S1 Nanosuspension particle diameter over 14 days of storage at room temperature (RT) or under refrigeration (4°C) (n = 3 per group). Data shown as mean ± SEM. *p < 0.05 compared to the starting diameter on Day 0 Figure S2. Moxifloxacin‐pamoate nanosuspension (MOX–PAM NS) showed superior prophylactic efficacy against ocular infections. The corneal swabs from Staphylococcus aureus infected rats from different groups were directly applied onto agar plates. A representative image of the agar plates from each group is shown to compare the bacterial burden qualitatively Figure S3. Histopathological evaluation of corneas obtained from untreated rats (infected control) or rats treated with Vigamox or MOX–PAM NS immediately after Staphylococcus aureus infection (images representative of n = 3 per group). Scale bar represents 50 μm and applies to all images. Figure S4. Histopathological evaluation of corneas obtained from untreated rats (infected control) or rats that received treatment beginning 24 h after Staphylococcus aureus inoculation with once daily Vigamox, three times daily Vigamox or once daily MOX–PAM NS for 3 days (images representative of n = 3 per group). Scale bar represents 50 μm and applies to all images [file BTM2-6-e10238-s001.docx]

**Supplementary information**


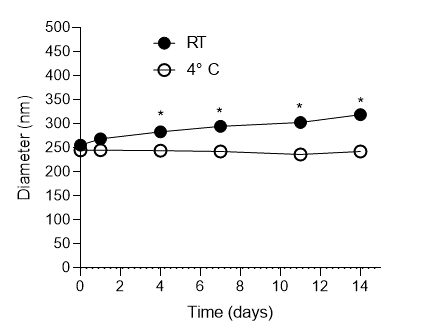


**Figure S1:** Nanosuspension particle diameter over 14 days of storage at room temperature (RT) or under refrigeration (4°C) (n = 3 per group). Data shown as mean ± SEM. **p* < 0.05 compared to the starting diameter on day 0.


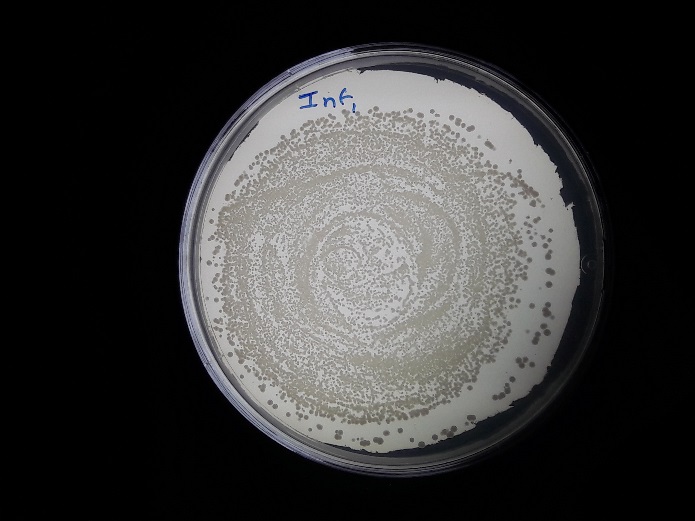

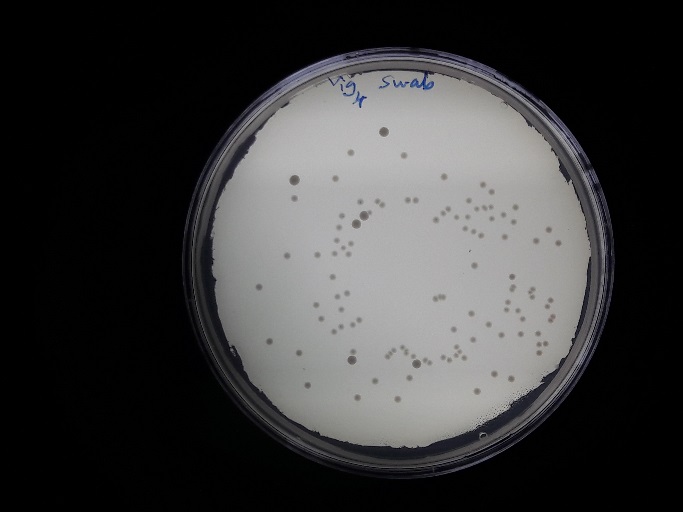

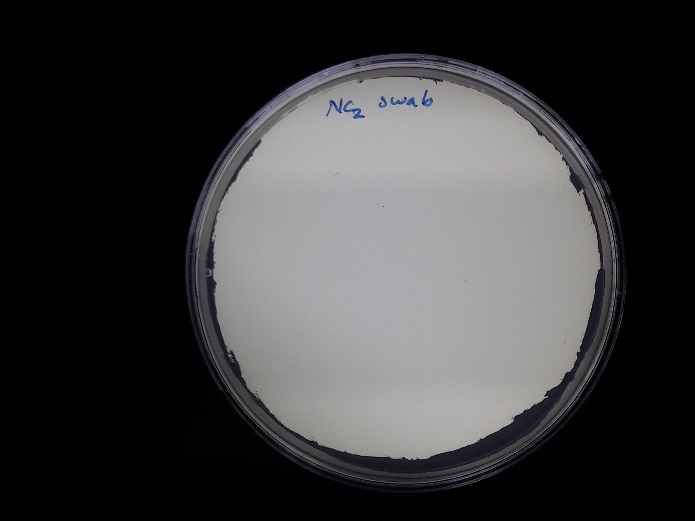


Infected Control

Vigamox^®^

MOX-PAM NS

**Figure S2:** Moxifloxacin-pamoate nanosuspension (MOX-PAM NS) showed superior prophylactic efficacy against ocular infections. The corneal swabs from *S. aureus* infected rats from different groups were directly applied onto agar plates. A representative image of the agar plates from each group is shown to compare the bacterial burden qualitatively.


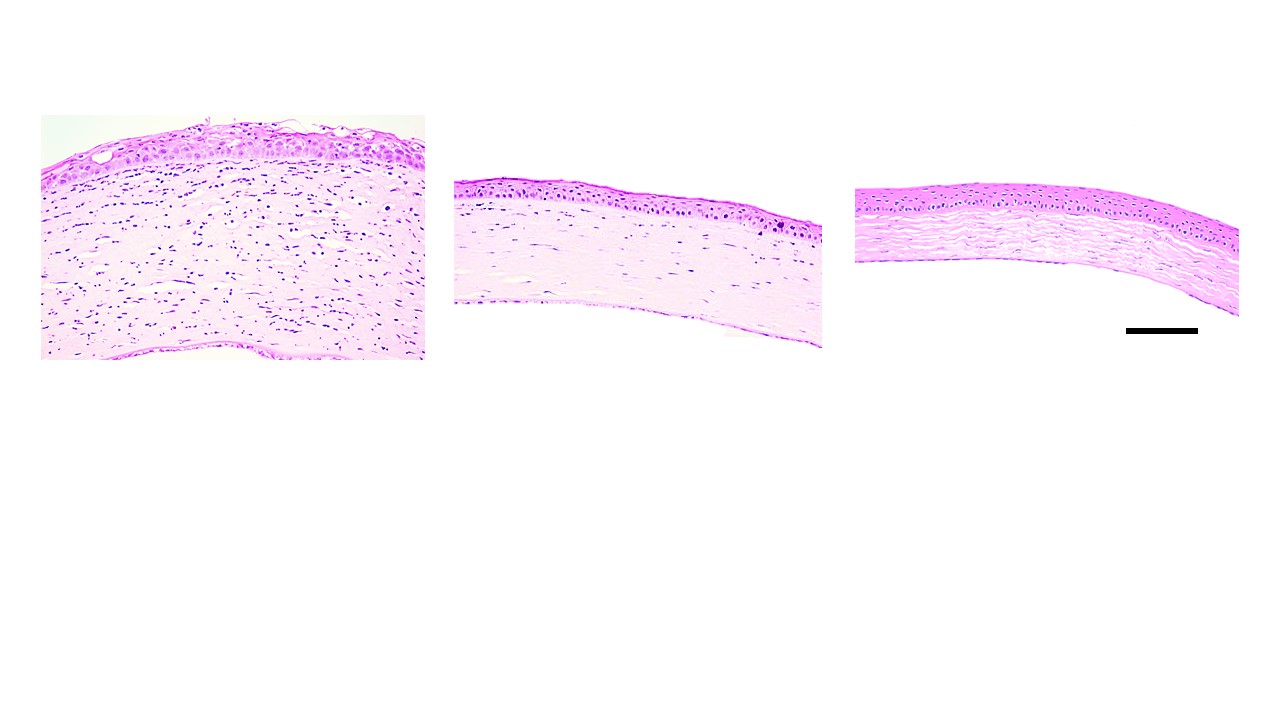


MOX-PAM NS

Vigamox

Infected Control

**Figure S3:** Histopathological evaluation of corneas obtained from untreated rats (infected control) or rats treated with Vigamox or MOX-PAM NS immediately after *S. aureus* infection (images representative of n = 3 per group). Scale bar represents 50 µm and applies to all images.


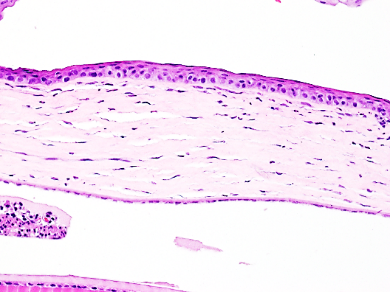

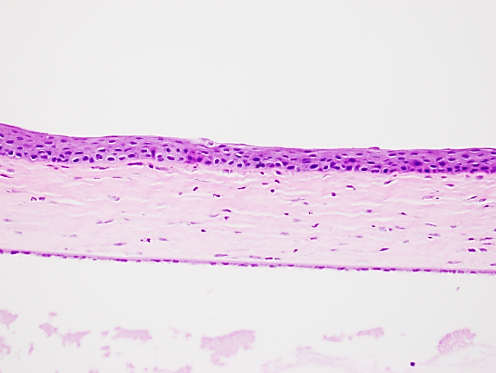

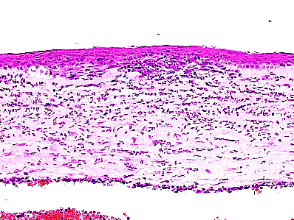

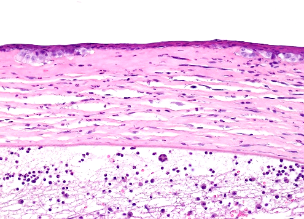


Infected Control

Vigamox once daily

Vigamox 3 times daily

MOX-PAM NS once daily

**Figure S4:** Histopathological evaluation of corneas obtained from untreated rats (infected control) or rats that received treatment beginning 24 h after *S. aureus* inoculation with once daily Vigamox, three times daily Vigamox or once daily MOX-PAM NS for 3 days (images representative of n = 3 per group).Scale bar represents 50 µm and applies to all images.
